# Highly efficient pollutant removal of graphitic carbon nitride by the synergistic effect of adsorption and photocatalytic degradation†

**DOI:** 10.1039/c7ra11467b

**Published:** 2018-02-13

**Authors:** Xueping Song, Qin Yang, Mengyun Yin, Dan Tang, Limei Zhou

**Affiliations:** Chemical Synthesis and Pollution Control Key Laboratory of Sichuan Province, China West Normal University Nanchong 637002 Sichuan China cwnuzhoulimei@163.com +86-817-2568081

## Abstract

Environmental remediation based on semiconducting materials offers a green solution for pollution control in water. Herein, we report a novel graphitic carbon nitride (g-C_3_N_4_) by one-step polycondensation of urea. The novel g-C_3_N_4_ material with a surface area of 114 m^2^ g^−1^ allowed the repetitive adsorption of the rhodamine B (RhB) dye and facilitated its complete photocatalytic degradation upon light irradiation in 20 min. This study provides new insights into the fabrication of g-C_3_N_4_-based materials and facilitates their potential application in the synergistic removal of harmful organic pollutants in the field of water purification.

## Abstract 2

A novel g-C_3_N_4_ with strong adsorption capability and efficient photocatalysis activity was prepared by heating urea through a facile method.

## Graphical abstract

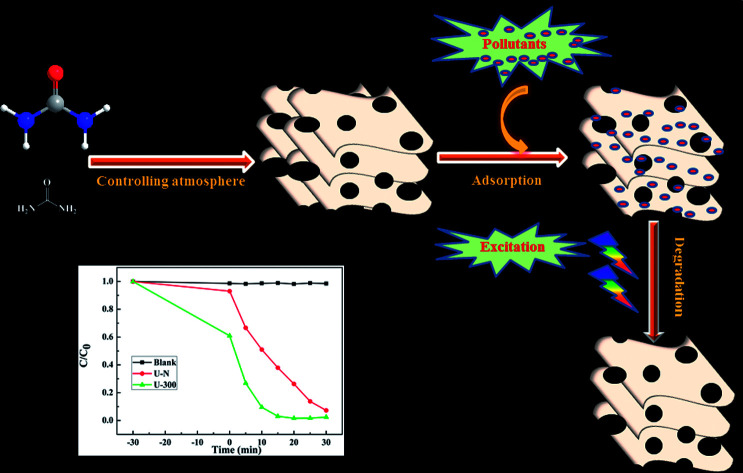

## 1. Introduction

The problems of environmental pollution have invigorated growing awareness all over the world.^1,2^ To date, a number of treatments, such as biodegradation,^3^ adsorption,^4^ and photocatalytic degradation,^5^ have been studied to remove organic pollutants in water. Photocatalysis based on the conversion of solar into chemical energy has been regarded to be one of the most promising technologies to remove environmental pollutants.^5,6^ Ever since the photocatalytic degradation of organic pollutants in aqueous suspensions has been reported by Carey *et al.* in 1976,^7^ there has been substantial development in the fabrication of highly efficient semiconductor-based photocatalysts.^8–10^ Recently, graphitic carbon nitride (g-C_3_N_4_) has been considered as the next generation photocatalyst and a step towards achieving sustainability for artificial photosynthesis and environmental remediation since the pioneering work has been reported in 2009.^11–16^ As a metal-free polymeric photocatalyst, g-C_3_N_4_ exhibits a number of excellent characteristics, such as facile synthesis, high chemical and thermal stability, reasonable cost, abundant and inexpensive building elements, appropriate electronic band structure for visible-light response, and flexible supermolecular networks for fine-tuning material properties, for photocatalysis.^13,17^ However, the practical applications of g-C_3_N_4_ are still hindered by the several obstacles and shortcomings, especially its low specific surface area, limited active sites, poor adsorption ability, and the serious aggregation observed during a photocatalytic process, of common bulk g-C_3_N_4_ prepared *via* the direct polycondensation of nitrogen-rich precursors.^17,18^ To overcome these drawbacks, many attempts, such as doping with heteroatoms,^19,20^ constructing heterostructures,^21,22^ fabricating copolymers,^23,24^ and thermal etching,^25,26^ have been dedicated towards improving the photocatalytic capability of g-C_3_N_4_. However, preparation of a highly active g-C_3_N_4_ material using a facile and eco-friendly strategy is still desirable. As is known, adsorption is also one of the most widely used methods to remove pollutants due to its convenient operation, low cost, and so on.^27–29^ However, adsorption cannot solve these problems drastically because organic pollutants just can be concentrated rather than degraded to non-polluting molecules. In addition, the materials need to undergo tedious desorption processes before being recycled.^30^ To combine photocatalysis with adsorption, a number of efforts have been devoted towards developing g-C_3_N_4_-based materials with strong adsorption. Chen prepared a g-C_3_N_4_/activated carbon composite photocatalyst with good efficiency in the photodegradation of phenol.^31^ An agar-C_3_N_4_ hybrid hydrogel photocatalyst with a 3D network structure was also prepared, and the hybrid hydrogel showed highly efficient pollutant removal ability *via* the synergistic effect of adsorption and photocatalytic degradation.^32^ In addition, polyaniline/carbon nitride nanosheets were shown to be highly excellent in removing organic pollutants on account of the cooperation of adsorptive preconcentration and a following photocatalytic oxidation reaction.^33^ Very recently, Panneri *et al.* reported bifunctional granules of carbon-doped g-C_3_N_4_ for the efficient removal of the antibiotic tetracycline.^34^ Inspired by these fruitful findings, we envisaged the preparation of a novel g-C_3_N_4_-based material with high photocatalytic activity and strong adsorption capability using a facile method.

**Fig. 1:**
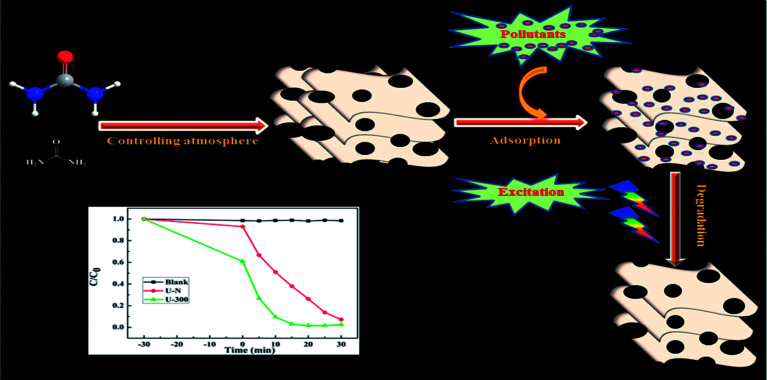
A schematic of the preparation of novel g-C_3_N_4_ and its excellent degradation efficiency *via* synergistic adsorption and degradation.

As illustrated, the characteristic properties and chemical structures of g-C_3_N_4_ are strongly affected by the reaction atmosphere through inducing disordered structures, defects, and carbon and nitrogen vacancies.^13^ In our previous study, we have successfully prepared modified g-C_3_N_4_ with high photocatalytic activity under a self-producing atmosphere. However, we found no enhancement in the adsorption capacity.^35^ In this study, we have developed novel g-C_3_N_4_ nanosheets from a urea precursor under a self-producing atmosphere by controlling the addition of N_2_, as shown in Fig. 1. When compared with conventional g-C_3_N_4_, the novel g-C_3_N_4_ nanosheets demonstrate an improved crystal structure, larger specific surface area, and more regular and homogeneous morphology. Moreover, the novel g-C_3_N_4_ material exhibited strong adsorption and efficient photocatalytic activity for the removal of rhodamine B (RhB) and could act as an excellent candidate for pollutant removal *via* the synergistic effect of adsorption and photocatalytic degradation. Our study provides new insights into the fabrication of g-C_3_N_4_-based materials and facilitates their potential application for the synergistic removal of various organic pollutants in the field of water purification.

## 2. Experimental

### 2.1. Sample preparation

The conventional g-C_3_N_4_ sample was prepared using a previously reported thermal polymerization method.^36^ In a typical procedure, the material was synthesized by heating 5 g of urea in a semi-closed alumina crucible under a flow of N_2_ gas to 550 °C for 4 h at a heating rate of 5 °C min^−1^ and then cooled down to room temperature. The product was obtained and ground into powder and denoted as U-N. The novel g-C_3_N_4_ material was prepared using a similar method as used for U-N with the exception that the addition of N_2_ at 300 °C was stopped. The obtained sample was denoted as U-300.

### 2.2. Catalyst characterization

The X-ray diffraction (XRD) patterns were obtained *via* a Rigaku Dmax/Ultima IV diffractometer using Cu Kα radiation (*λ* = 1.5418 Å) to evaluate the crystal structure and phase purity. Fourier transform infrared (FTIR) spectra were obtained using a Perkin-Elmer model 2000 FTIR spectrophotometer. Scanning electron microscopy (SEM) images were acquired using an FEI QUANTA F250 scanning electron microscope. X-ray photoelectron spectroscopy (XPS) measurements were carried out using an ESCALAB 250 Xi with a high-performance Al monochromatic source (*hν* = 1486.6 eV, 150 W). All binding energies were calibrated by setting the C 1s peak to 284.8 eV for surface adventitious carbon, and the elemental compositions were determined from the peak area ratios after correction for the sensitivity factor for each element. Brunauer–Emmett–Teller (BET) surface area measurements were conducted using the N_2_ adsorption–desorption isotherms obtained at 77 K using Quantachrome Instruments version 3.0. UV-vis diffuse reflectance spectra (DRS) were obtained *via* a Shimadzu UV-3600 spectrophotometer using BaSO_4_ as the reference sample. The photoluminescence (PL) spectra of the photocatalysts were obtained using a Varian Cary Eclipse spectrometer with the excitation wavelength of 350 nm. The RhB adsorption and degradation were monitored by a Shimadzu UV-2550 UV-vis spectrophotometer at definite time intervals.

### 2.3. Adsorption and photocatalytic activity

The adsorption capacities and photocatalytic activities of the synthesized samples were evaluated using the adsorption and photodegradation of RhB at ambient temperature in air under magnetic stirring. To find out the differences in the adsorption performance, 25 mg of the photocatalysts was dispersed in a RhB solution (10 mg L^−1^). The resulting suspension was first sonicated for 10 min and then stirred continuously in the dark for 50 minutes. Aliquots were taken every 10 minutes to measure the adsorbed concentration using a UV-vis spectrophotometer. The photocatalytic activities of the synthesized samples were then evaluated through degradation of RhB under irradiation. The UV-visible light has been provided by a 70 W metal halide, which is often used in photocatalytic tests conveniently.^37–39^ In a typical photocatalytic experiment, 25 mg of photocatalyst was added to 50 mL of RhB aqueous solution (10 mg L^−1^) at room temperature. Prior to irradiation, the mixed suspension was first sonicated for 10 min and then magnetically stirred for 20 min in the dark to obtain an adsorption–desorption equilibrium. At given intervals during irradiation, about 3 mL of sample was taken out from the reaction system and centrifuged to remove the photocatalyst powders. The absorbance of RhB in the supernatant was measured using a UV-vis spectrophotometer at 554 nm. The efficiency of degradation was calculated by *C*/*C*_0_, where *C* is the concentration of remaining dye solution at time *t* and *C*_0_ is the initial concentration.

The stability of U-300 was investigated using recyclability studies. After each cycle, the catalyst was obtained by centrifugation, washed with distilled water, and dried at 60 °C overnight. Then, the recovered catalyst was directly used for the next cycle of photocatalytic degradation of RhB as abovementioned.

## 3. Results and discussion

### 3.1. Structural characteristics

**Fig. 2:**
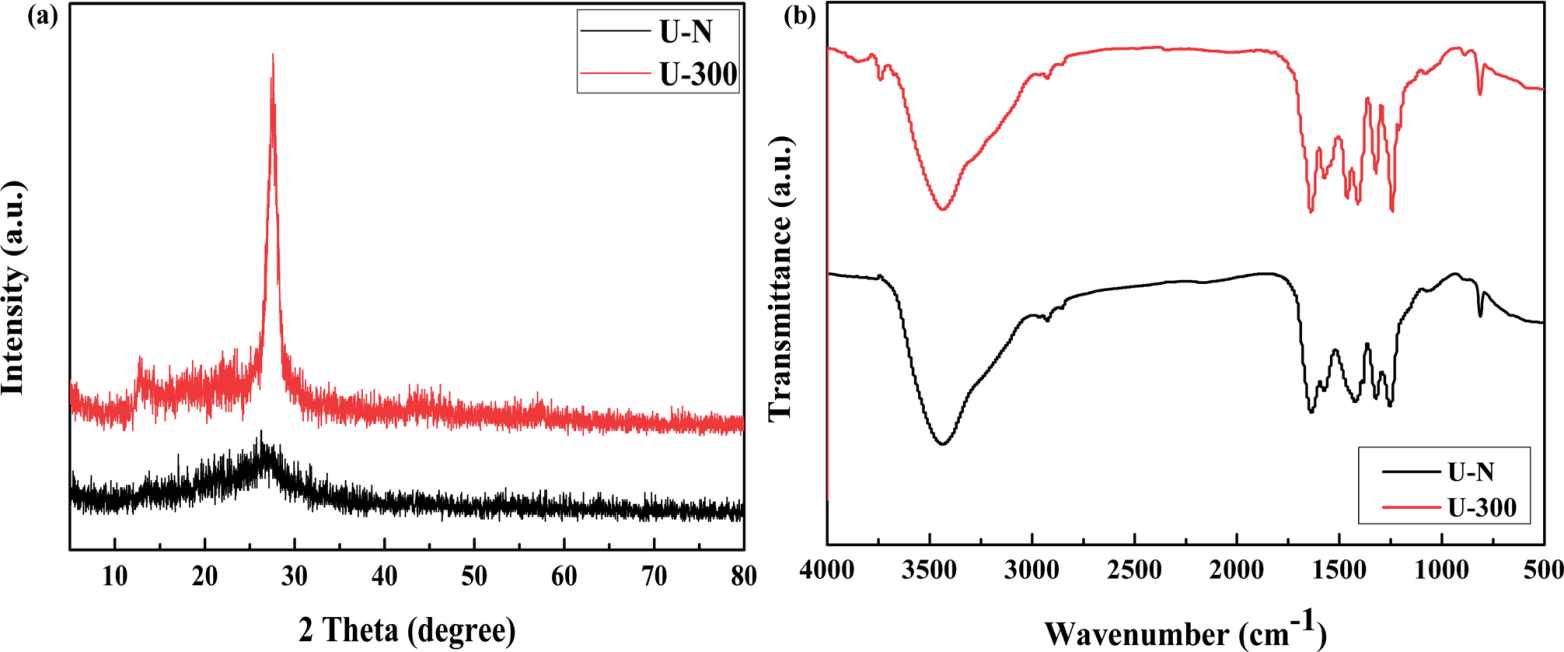
The (a) XRD patterns and (b) FT-IR spectra of the two samples.

The XRD patterns of the common and novel g-C_3_N_4_ are displayed in Fig. 2a. The two materials show distinctively different XRD patterns. U-300 shows two typical diffraction peaks at around 12.8° and 27.6°, as reported previously,^11–13^ which are due to the in-plane structural packing motif and periodic stacking of layers along the *c*-axis, respectively. However, the two sharp peaks become very weak in the U-N pattern, which demonstrate the absence of long-range order in the atomic arrangements.^40,41^ We may infer the multiple effects of too many gas bubbles, which are produced during thermal condensation of urea, and the additional N_2_ significantly disrupts the long-range atomic order in both the perpendicular and parallel directions to the g-C_3_N_4_ layers. After stopping the addition of N_2_, the self-producing atmosphere originates from the interior of the reaction system. The condition of polycondensation was more homogeneous; thus, we obtained an improved crystal structure. Cui *et al.* have reported that the well-condensed crystallinity of g-C_3_N_4_ indicates a larger surface area and improved transport of photogenerated carriers in its network.^41^ It is reasonable to expect the U-300 sample may show enhanced photocatalytic activity. Moreover, the main peak was slightly shifted to higher angles: U-N at 27.1° and U-300 at 27.6°, corresponding to a reduction in the stacking distance of the graphitic-layered structure. Merschjann *et al.* have shown that electronic transport is predominantly perpendicular to the sheets in g-C_3_N_4_.^42^ Thus, the decreased interlayer distance may be in favor of charge transport and thus improve the photocatalytic activity.

The microstructures of the conventional and novel g-C_3_N_4_ samples were further revealed using Fourier transform infrared (FT-IR) spectroscopy, which was sensitive to the local (or short-range) structure of the materials (Fig. 2b). The peak at approximately 810 cm^−1^ originates from the characteristic breathing mode of the tri-s-triazine units.^43^ The bands in the range from *ca.* 1645 to 1235 cm^−1^ can be attributed to the typical stretching modes of C–N heterocycles. The broad absorption bands located in the range from 3700 to 3000 cm^−1^ are assigned to the stretching vibration of N–H and O–H bonds, associated with the uncondensed amino groups and surface-bonded H_2_O molecules, respectively.^41,43^ We can see that the FTIR spectra of the two g-C_3_N_4_ materials show similar characteristic vibrational peaks; however, the peak intensity and stretching modes of the skeletal U-300 network are enhanced and better resolved probably due to the better organization of the conjugated system, which are also in agreement with the previously reported results.^41,44,45^

**Fig. 3:**
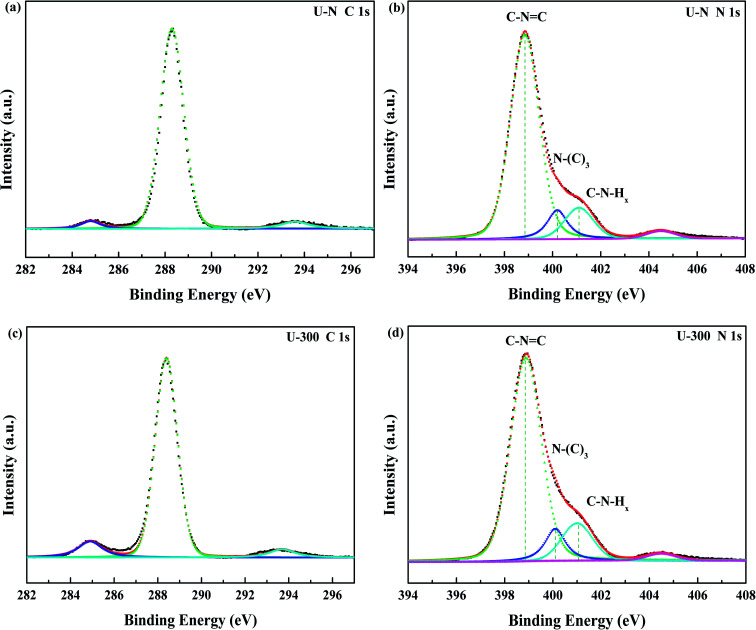
The high-resolution N 1s XPS spectra and C 1s XPS spectra of the two samples.

The compositions and surface chemical states of the two different samples were studied using X-ray photoelectron spectroscopy (XPS). In the XPS survey spectra (Fig. S2, ESI†), only C, N, and O were detected. The very weak O 1s peak may be due to the surface absorbed H_2_O or O_2_.^46,47^ Both samples exhibited similar C 1s and N 1s spectra without any significant peak shifts; this indicated similar chemical states. The deconvoluted C 1s spectra (Fig. 3a and c) showed three peaks at 284.8, 288.4, and 293.6 eV, which corresponded to surface carbon contamination, sp^2^ hybridized carbon of the tri-s-triazine rings, and π → π* satellite band.^48^ The N 1s XPS spectra (Fig. 3b and d) can be fitted into four peaks. The main peaks at 398.9 eV, 400.1 eV, and 401.1 eV correspond to the sp^2^-hybridized nitrogen in the heterocycle (C–N=C), tertiary N in the form of N(–C)_3_, and uncondensed amino functional groups (NH_2_ or NH),^45,49^ respectively. A weak peak at 404.5 eV of N 1s was assigned to the π → π* satellite band, which was very much like the satellite component for its C 1s signal.^48^ The absorption associated with the different nitrogen moieties identifies the defect types to some degree, as well as the uncondensed amino groups.^26,49^ The N-associated species were quantified using the deconvoluted N 1s spectra (Table 1). A larger value of NH_*x*_/N(–C)_3_ demonstrates the smaller degree of polymerization and more uncondensed amino groups. It can be found that U-300 shows a larger value of NH_*x*_/N(–C)_3_. The results illustrated that after stopping the entrance of N_2_, the pyrolysis-generated self-producing atmosphere influenced the process of thermal polymerization, resulted in a decreased degree of polymerization and a large number of amino groups, which concurred with our FTIR results.

**Table 1:** The relative content of the various nitrogen species obtained from the N 1s XPS data (%)

Sample	C=N–C	N(–C)_3_	NH_*x*_	NH_*x*_/N(–C)_3_
U-N	0.781	0.100	0.120	1.200
U-300	0.764	0.101	0.134	1.327

### 3.2. Textural properties

**Fig. 4:**
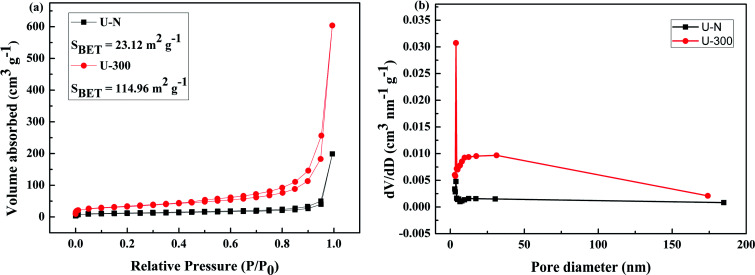
The (a) N_2_ adsorption–desorption isotherms and (b) BJH pore-size distribution curves obtained for two different samples.

The pore structures and BET surface areas of two different samples were obtained using the N_2_ adsorption–desorption measurements conducted at 77.4 K. As shown in Fig. 4a, U-300 exhibits type IV isotherms with an extremely high adsorption capacity in the high relative pressure region (*P*/*P*_0_: from 0.8 to 1); this indicates the presence of abundant mesopores and macropores.^45,50^ The BET surface area of U-300 was calculated to be 114.96 m^2^ g^−1^, which was about 5 times that of U-N (23.12 m^2^ g^−1^). To further analyze the pore structures, the pore size distribution using the BJH method (Fig. 4b) provided the pore sizes. Accordingly, a sharp peak at about 3.8 nm and a broad distribution in the range from 10 to 170 nm were identified in the pore size distribution curve for U-300. The higher BET surface area and larger pore size distribution may be caused by the effective prevention of the aggregation of the g-C_3_N_4_ nanosheets by the self-producing atmosphere. The large surface area and pore volume can provide more reactive sites and more edge structures and may effectively adsorb more reactants and be conducive to mass transfer and charge carrier transfer during the photocatalytic process.^45,50^

### 3.3. Morphology information

**Fig. 5:**
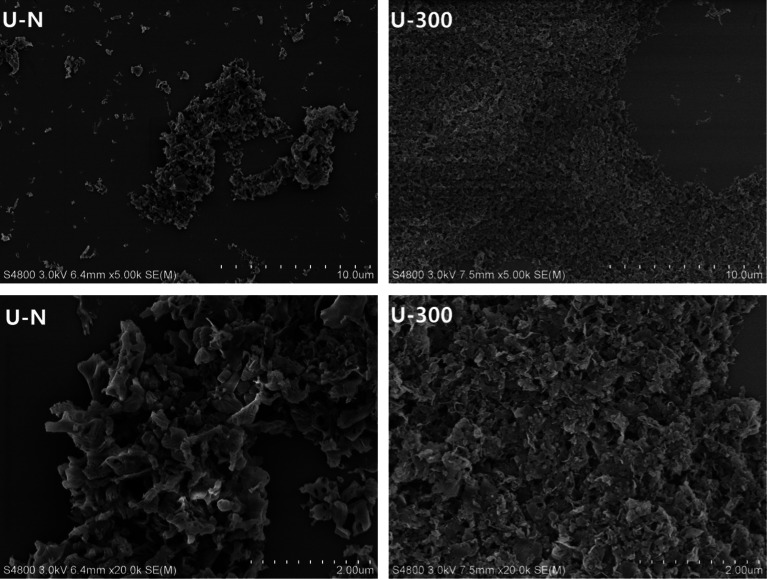
The SEM patterns of the two different samples.

The typical SEM images of the as-prepared samples are illustrated in Fig. 5. As can be seen, the U-N sample displays disorderly stacked irregular clusters and is mainly composed of interconnected thin layers with some pores that may result from the gas bubbles formed during the pyrolysis of urea. Contrary to U-N, U-300 exhibits an obviously layered platelet-like surface morphology. Furthermore, the images of U-300 display smaller particle sizes, better dispersion, and a more regular and homogeneous morphology. Based our previous study, N_2_ can offer an inert, sole positive atmosphere before polycondensation, whereas after stopping the addition of N_2_, the atmosphere of polycondensation originates from the interior of the reaction system. The conditions for polycondensation are more homogeneous; thus, we obtain a regular and homogeneous morphology, which is in agreement with our XRD and FTIR results. Obviously, this is a facile way to tune the microstructures of g-C_3_N_4_.

### 3.4. Optical properties

**Fig. 6:**
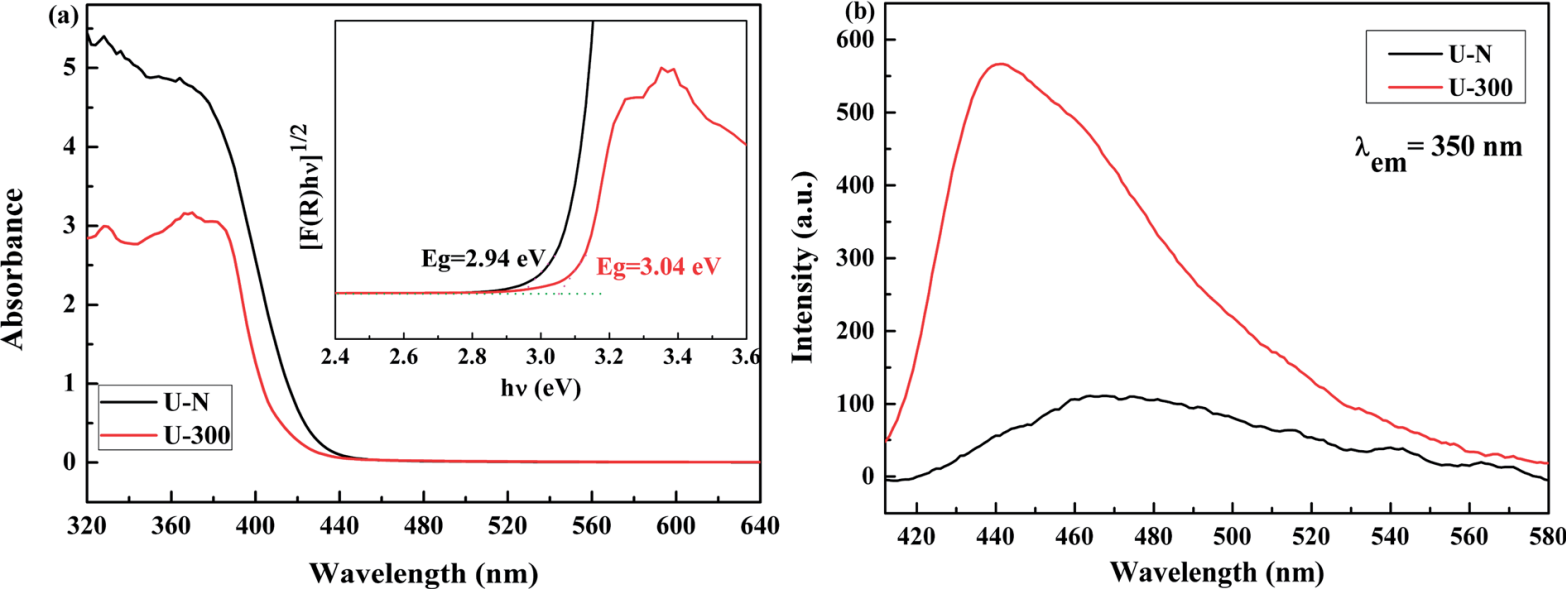
The (a) UV-visible diffuse reflectance spectra and (b) PL emission spectra at an excitation wavelength of 350 nm of the two different samples.

The electronic band structures and photoelectric properties of the two materials were analyzed by UV-vis diffuse reflectance spectroscopy (DRS) (Fig. 6a) and photoluminescence spectroscopy (PL) (Fig. 6b). The UV-vis DRS spectra indicate that the absorption edge of the novel g-C_3_N_4_ sample displays a remarkable blue shift. This result is consistent with the pale yellow color (Fig. S3†) and indicates the low degree of polycondensation. Accordingly, the electronic band gaps derived from the Tauc plots (Fig. 6a) are 2.94 eV for U-N and 3.04 eV for U-300. The band gap of U-300 was widened by 0.10 eV as compared to that of U-N. The increase in the band gap by 0.10 eV improves the redox ability of the charge carriers generated in the CN nanosheets.^40^ This result was further confirmed by the blue-shift in its fluorescence emission peak in Fig. 6b; this could be attributed to the quantum confinement effect.^41,45^

The photoluminescence emission (PL) spectra originating from the recombination of free charge carriers are important to reveal the separation, migration, and recombination of the photogenerated charge carriers. As shown in Fig. 6b, all the excitation spectra were monitored at room temperature with an excitation wavelength of 350 nm and match well with UV-vis diffuse reflectance spectra. Note that U-N has a low PL emission peak. According to the literature, surface defects may act as e^−^/h^+^ recombination sites that result in increasing the non-radiative recombination rates and reduce the free carrier concentration; this leads to a decreased radiative PL intensity.^47^ For U-300, the enhanced PL intensity can be attributed to the improved crystal structure.^41,47,51,52^ Furthermore, the blue-shifted wavelength of the fluorescence emission peak observed for U-300 illustrates the smaller degree of polycondensation as compared to the case of U-N and the presence of sub-gap defects in the material.^47^

**Fig. 7:**
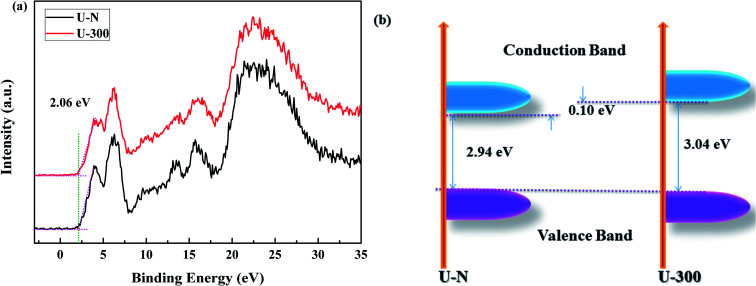
The (a) VB XPS spectra and (b) electronic band structures of the two different samples.

The XPS valence band (VB XPS) spectra were analyzed to investigate the band edges of the two different samples. In Fig. 7a, it can be seen that the samples have the same VB of ∼2.06 eV. When combined with the UV-vis DRS results, we found that the CB of U-300 was up-shifted; this indicated its stronger redox abilities.^41^

### 3.5. Adsorption and photocatalytic performance

**Fig. 8:**
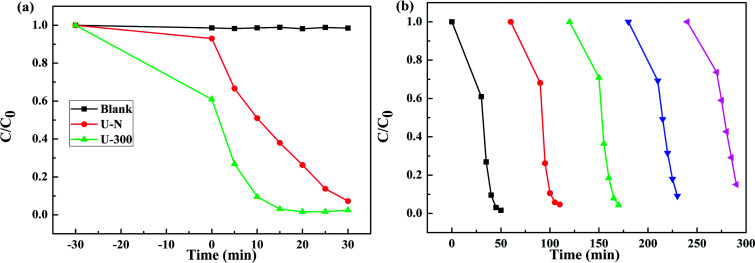
(a) The adsorption and photocatalytic degradation of RhB. (b) The cycling tests for the adsorption and photocatalytic degradation of RhB using U-300.

Fig. 8a presents the change in the concentration of RhB with time for the two samples. RhB does not undergo self-photolysis as its concentration remains unchanged with time. After reaching an adsorption equilibrium under the same conditions, the U-300 material showed a higher percentage of adsorption (40%) as compared to U-N (8%). The adsorption of RhB was monitored for 60 min, and it was observed that the adsorption equilibrium was reached within 20 min (Fig. S4 and S5†). The porous morphology and large surface area of U-300 induced high rates of adsorption, and the adsorbed RhB was degraded by more than 90% within 15 min under UV-visible light illumination (Fig. 8a). The U-300 sample was tested through five consecutive trials to test its reusability and stability. There was hardly any loss of activity in the adsorption and photocatalytic performance of U-300 upon prolonging the reaction time (Fig. 8b). To further test the photocatalytic performance, half mass of U-300 also showed excellent photocatalytic degradation (Fig. S8†). To confirm the universality of the catalyst, we have studied the photocatalytic degradation of tetracycline hydrochloride (TC-HCl), which is a common antibiotic and colorless pollutant. The results also verified the excellent ability of U-300 to remove organic pollutants in water (Fig. S9†). Based on the abovementioned experimental results, U-300 can be regarded as a stable high-performance photocatalyst for the photodegradation of organic pollutants, possessing great prospects in environmental protection.

**Fig. 9:**
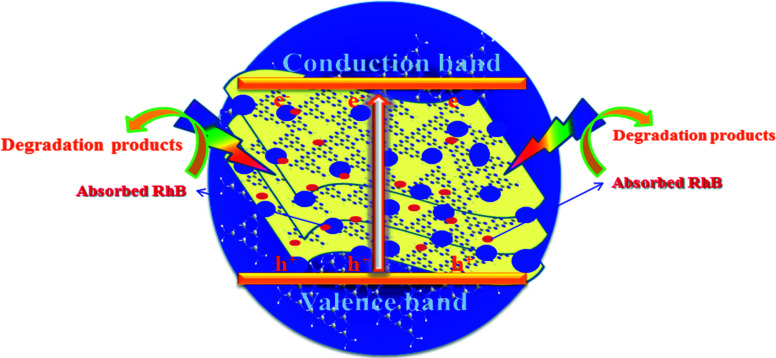
A schematic of the synergistic adsorption and photocatalytic degradation processes.

As shown in Fig. 9, the mechanism of the adsorption-enrichment and photocatalytic degradation synergistic effect is proposed. At first, the organic contaminant was adsorbed and enriched onto the surface of the novel g-C_3_N_4_ nanosheets. The large surface area, small aggregation, and porous structure of the novel g-C_3_N_4_ nanosheets provide more reactive sites and more edge structures and might effectively adsorb more reactants.^50,53^ Furthermore, the improved graphitic-like structure and shorter interlayer distance may accelerate charge transport and thus improve the photocatalytic activity.^41,42^ Finally, acting as an absorbent as well as photocatalyst, after being absorbed onto the surface of the novel g-C_3_N_4_ nanosheets, the organic contaminant was then degraded *in situ* under light irradiation to realize the synergistic effect of adsorption and photocatalysis. Overall, the adsorbed organic contaminants as well as the organic contaminants retained in solution were subsequently degraded under light irradiation.

## 4. Conclusions

In conclusion, a novel g-C_3_N_4_ with strong adsorption capability and efficient photocatalytic activity was prepared by heating urea *via* a facile method. The self-producing atmosphere during the calcination process induced the condensation process, offering extra structural control for the synthesis of the g-C_3_N_4_ networks. Compared to that of the conventional g-C_3_N_4_, the texture of the novel g-C_3_N_4_ nanosheets was optimized to have higher surface area, an improved crystal structure, more homogeneous morphology, and smaller particles. The large surface area, porous structure, well-condensed crystallinity, and enlarged band gap are proposed to be primarily responsible for the enhanced adsorption and photocatalytic activity of U-300. Overall, this study demonstrates a convenient route to synthesize a novel g-C_3_N_4_ catalyst with attractive photocatalytic activity without the need of any complex modification steps.

## Conflicts of interest

There are no conflicts to declare.

## Supplementary Material

RA-008-C7RA11467B-s001
