# Supplementary material for: Highly efficient pollutant removal of graphitic carbon nitride by the synergistic effect of adsorption and photocatalytic degradation
Source: RSC Adv. 2018 Feb 13;8(13):7260–8. doi: 10.1039/c7ra11467b (PMC9078411; doi:10.1039/c7ra11467b)
Supplement: RA-008-C7RA11467B-s001 [file RA-008-C7RA11467B-s001.pdf]

Electronic Supplementary Material (ESI) for RSC Advances.
This journal is © The Royal Society of Chemistry 2018

Supporting Information

Highly efficient pollutants removal of graphitic carbon nitride by synergistic effect of adsorption and photocatalytic degradation

Xueping Song, Qin Yang, Mengyun Yin, Dan Tang and Limei Zhou*

Chemical Synthesis and Pollution Control Key Laboratory of Sichuan Province, China West Normal University, Nanchong 637002, Sichuan, China.

Corresponding Author:*Tel: +86-817-2568081. E-mail: cwnuzhoulimei@163.com.

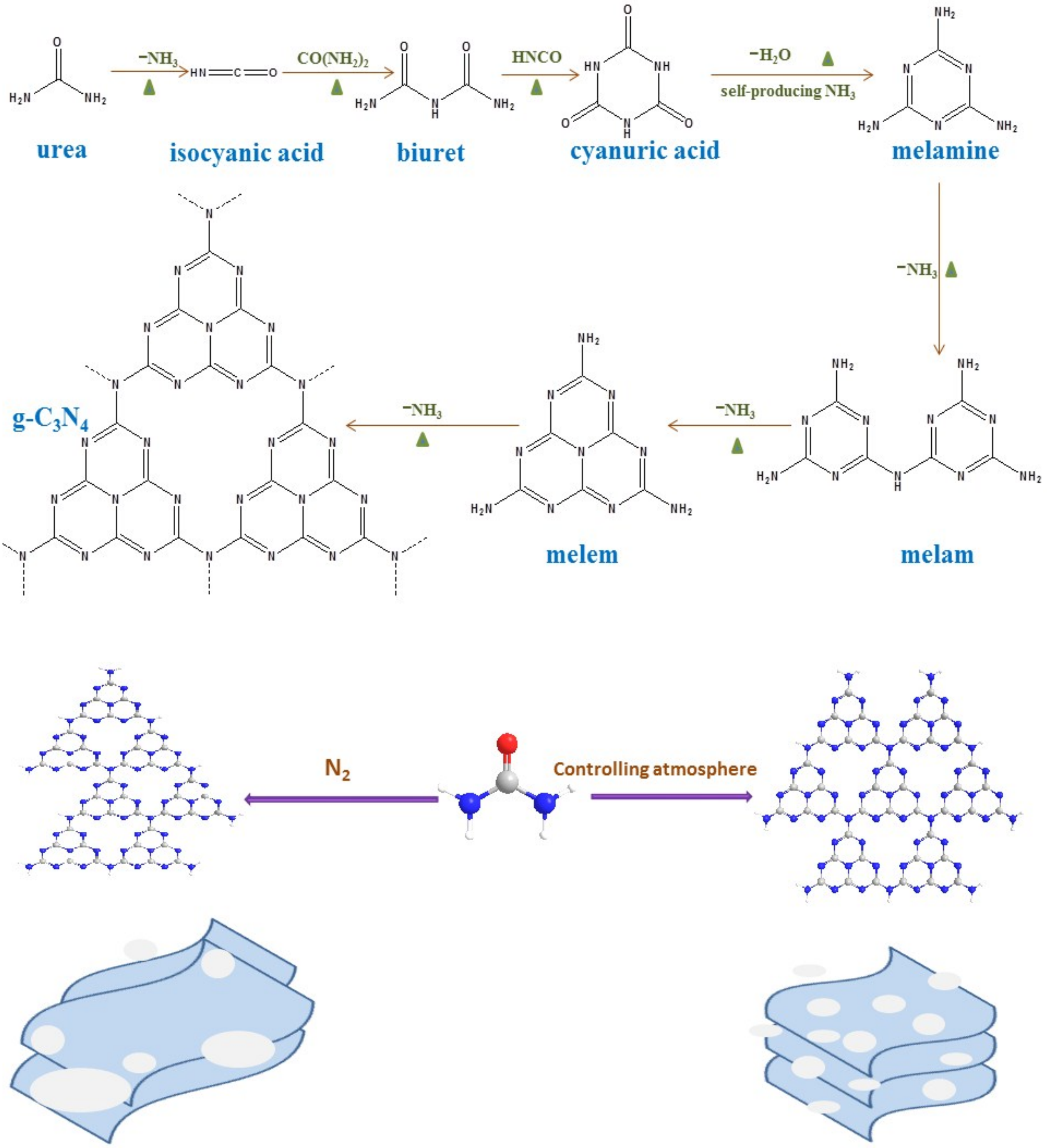

Fig. S1. Proposed mechanism of the reaction path for self-condensation of urea into g-C_3N_4.

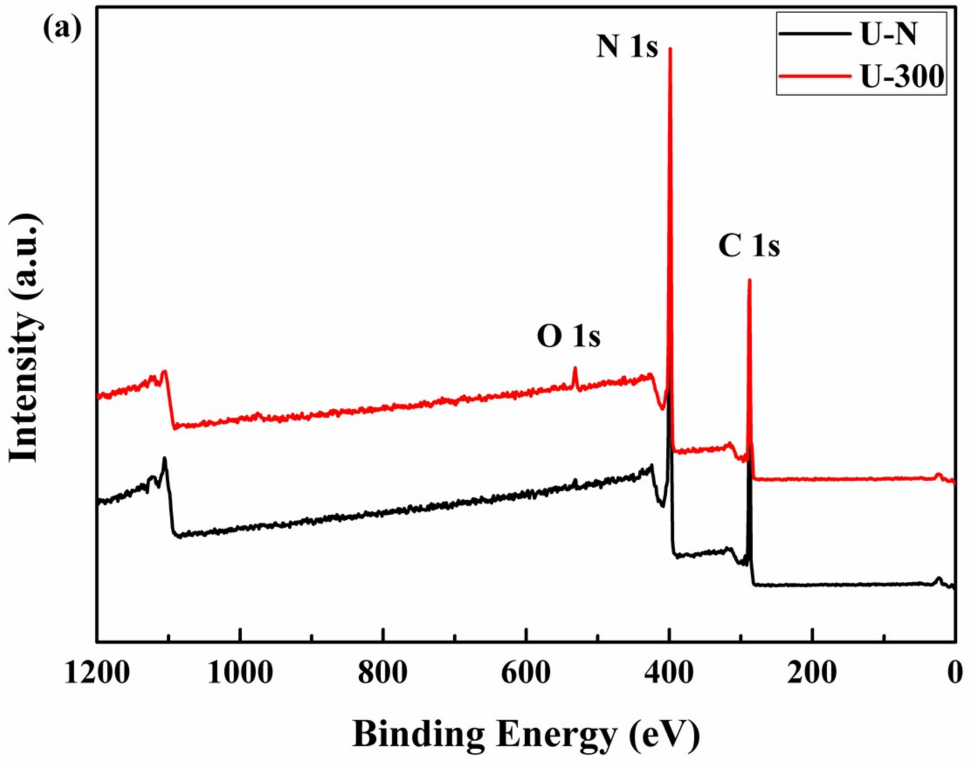

Fig. S2. XPS survey spectrum of different g-C_3N_4 samples.

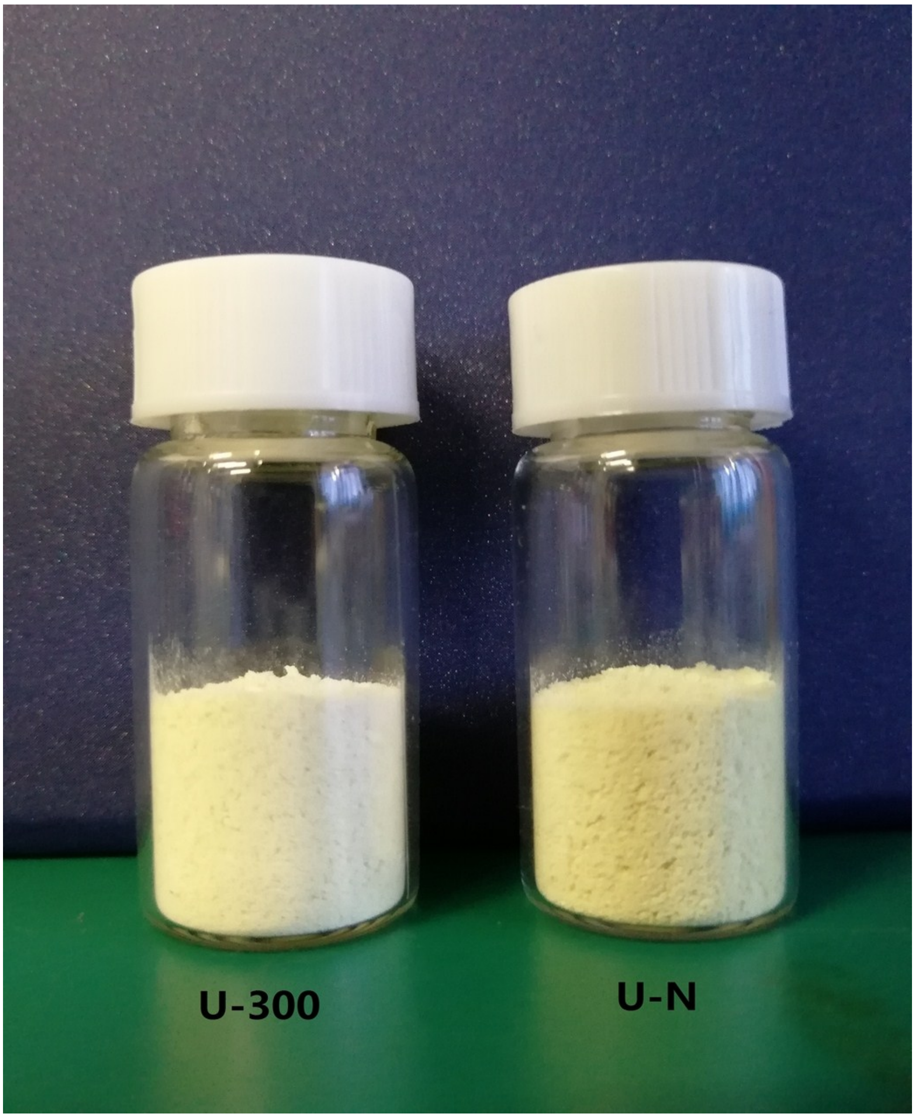

Fig. S3. Photograph of different g-C_3N_4 samples.

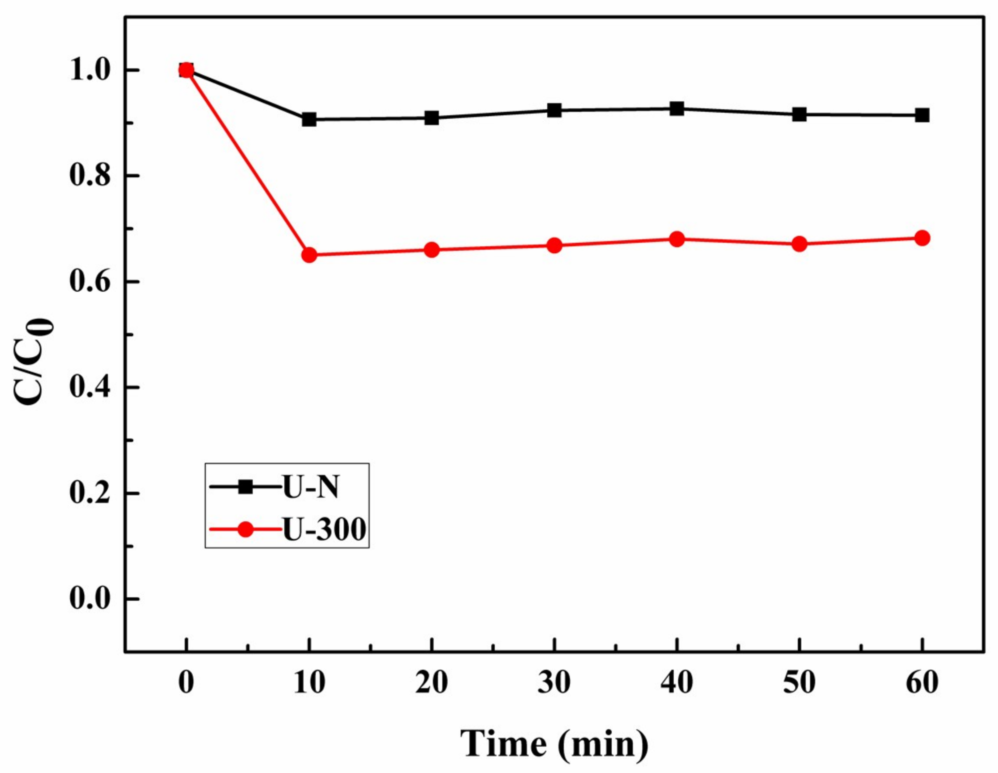

Fig. S4. Adsorption of RhB (10 mg/L, 50 mL) using different g-C_3N_4 samples (25 mg) in dark.

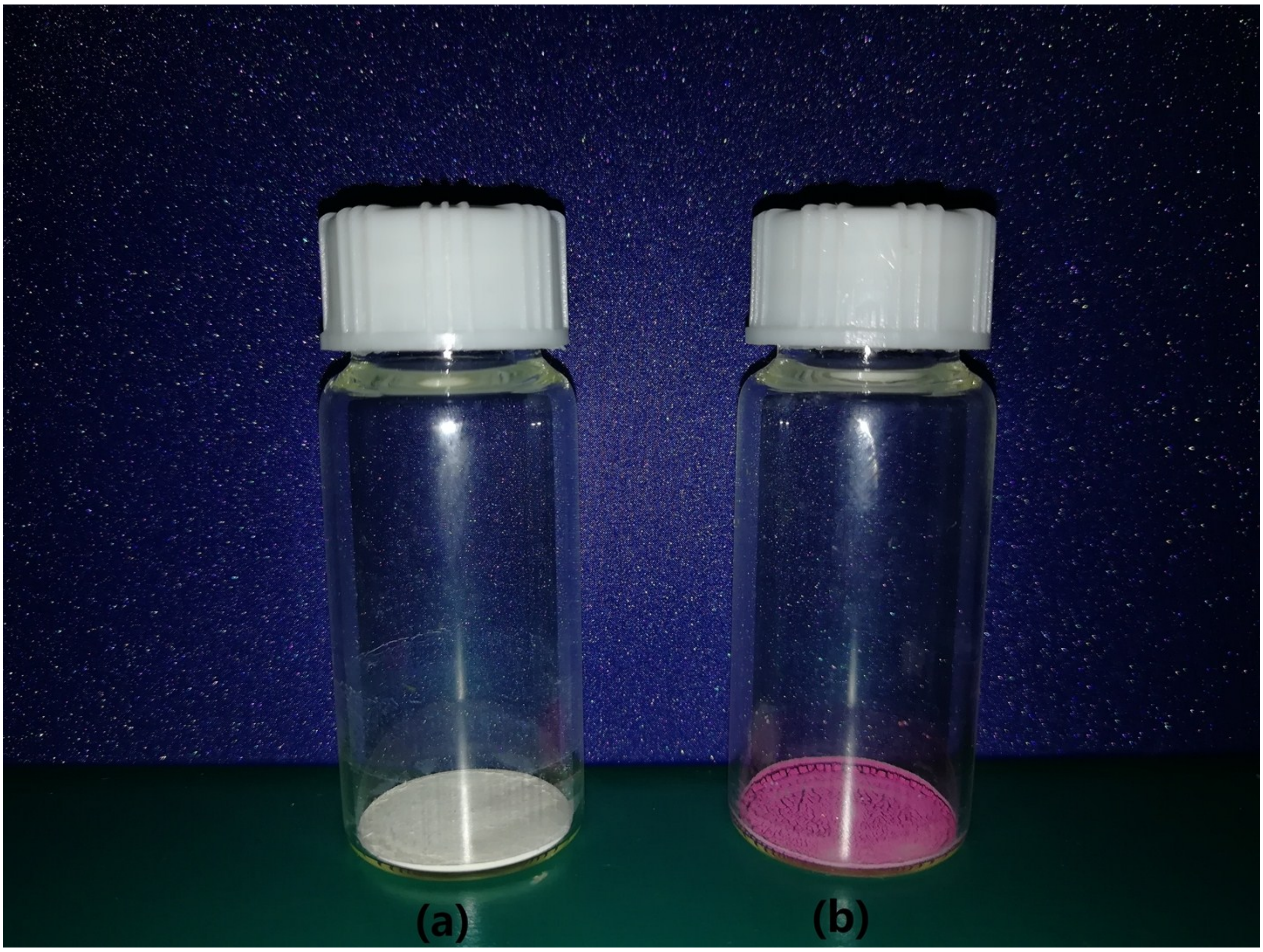

Fig. S5. Photograph of U-N (a) and U-300 (b) after adsorption of RhB.

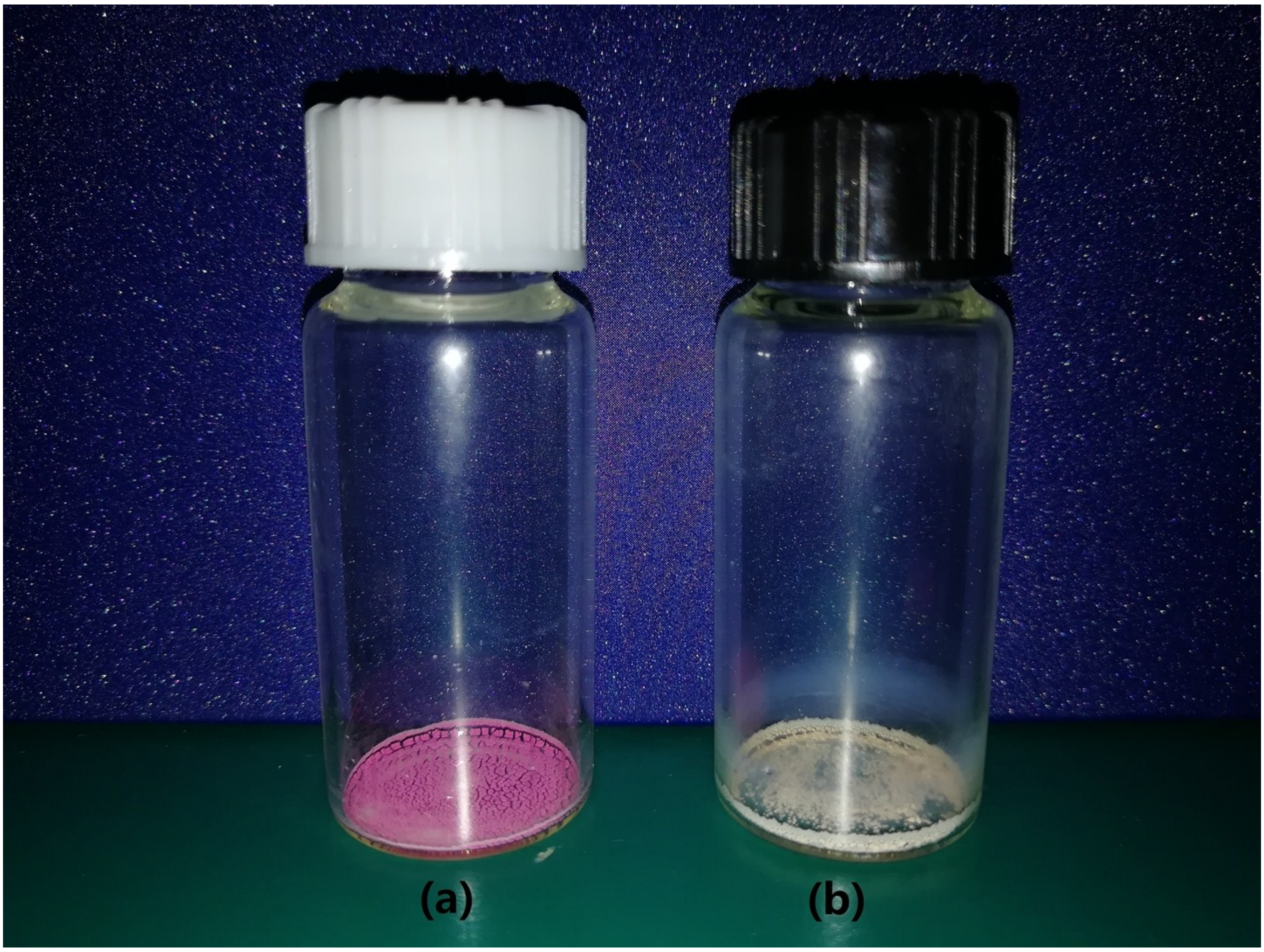

Fig. S6. Photograph of U-300 after adsorption (a) and degradation (b) of RhB.

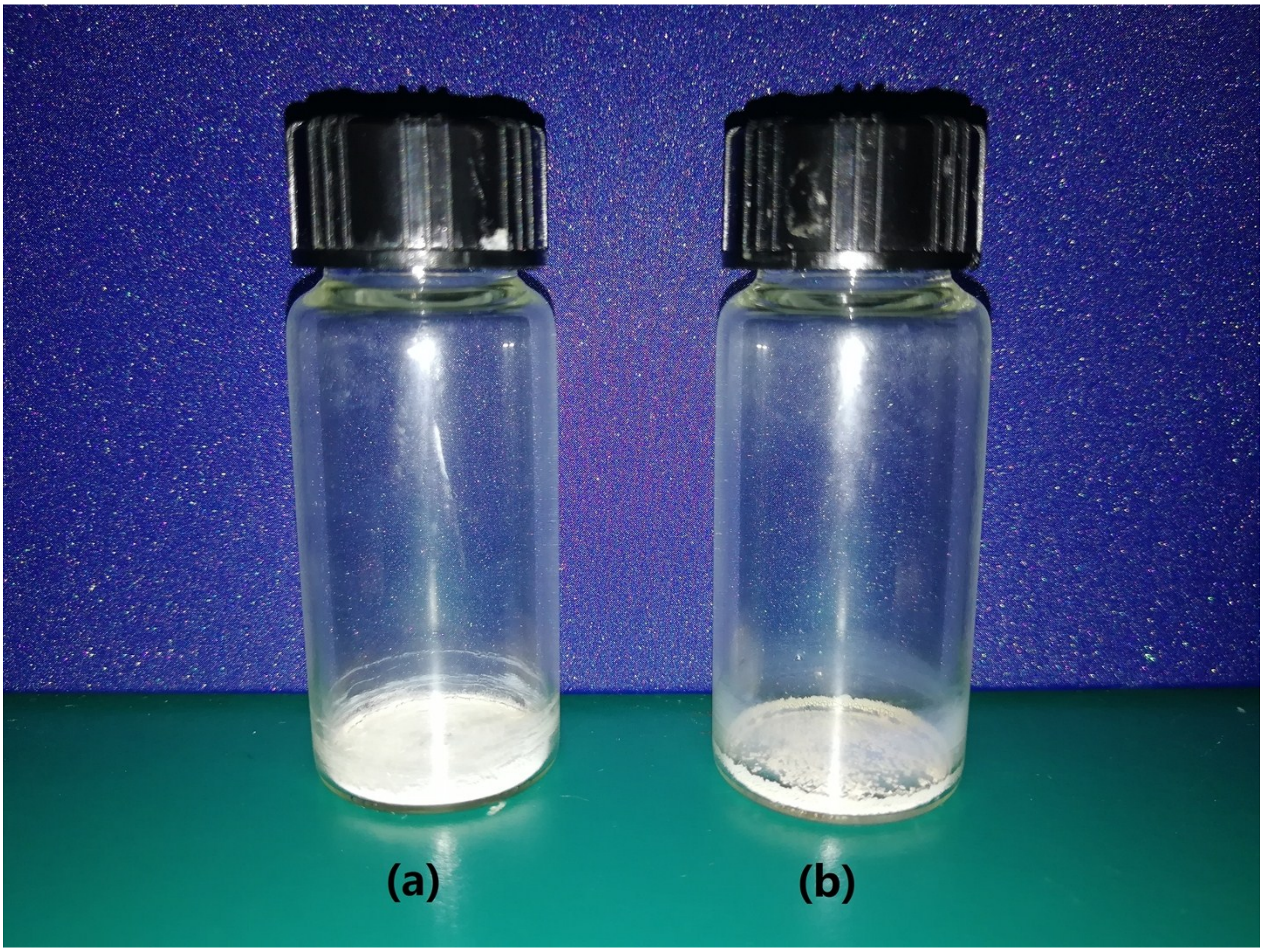

Fig. S7. Photograph of U-N (a) and U-300 (b) after degradation of RhB.

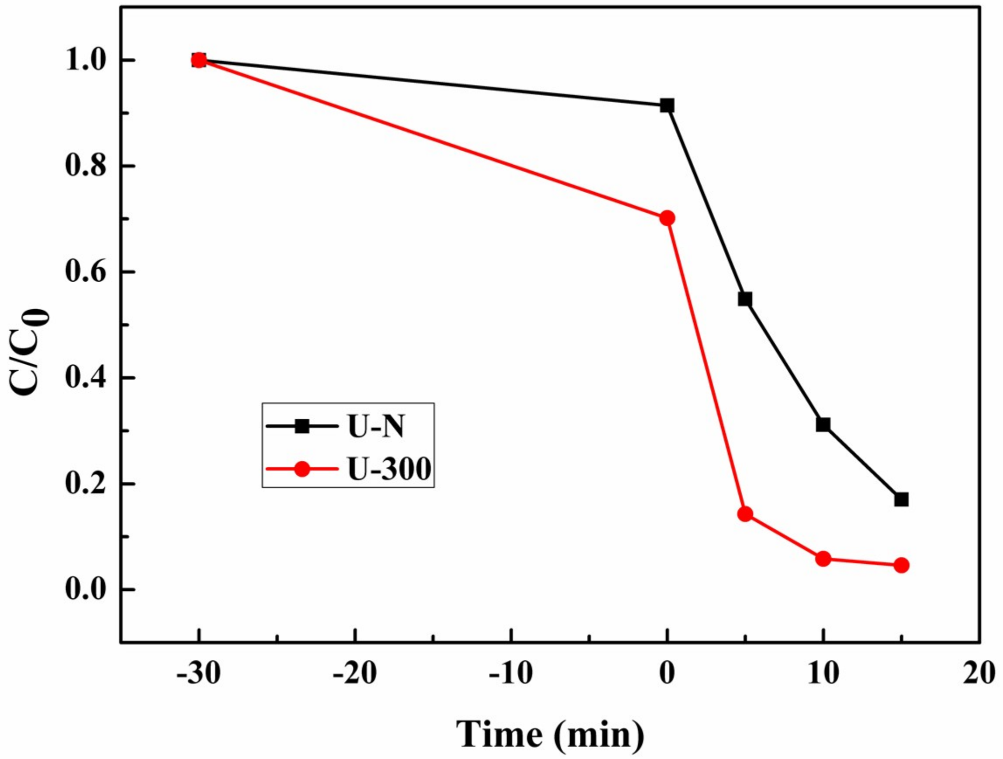

Fig. S8. Adsorption with photocatalytic degradation of RhB (5 mg/L, 50 mL) using different g-C_3N_4 samples (12.5 mg).

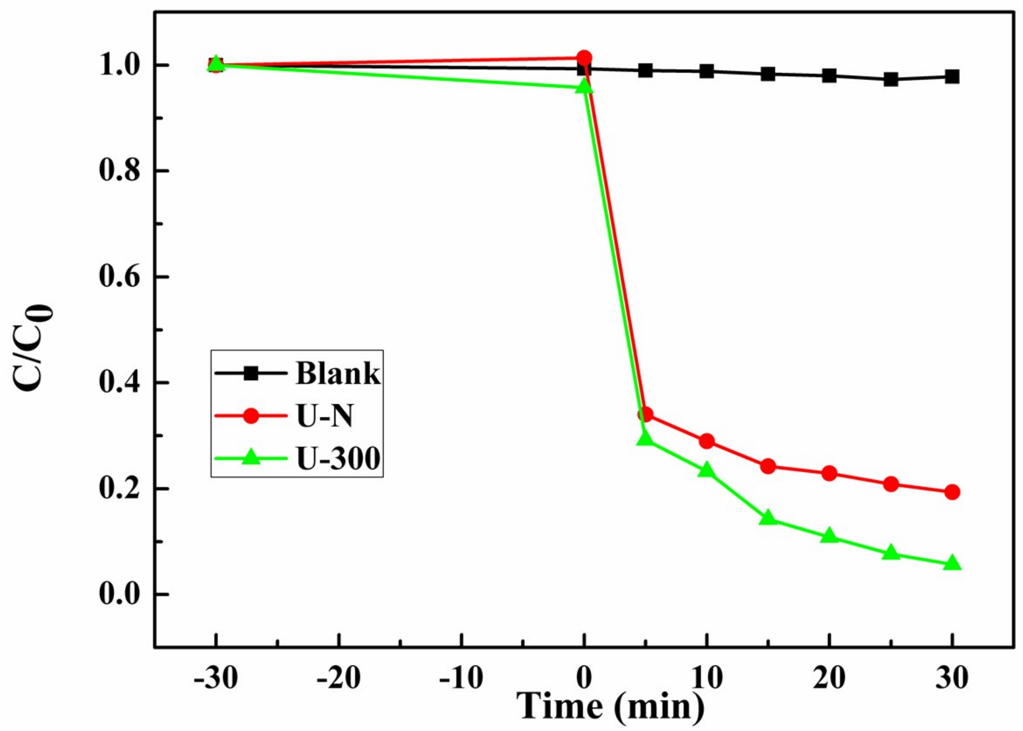

Fig. S9. Photocatalytic degradation of TC-HCl (20 mg/L, 50 mL) using different g-C_3N_4 samples (25 mg).
